# Supplementary material for: Disruption of the Chitin Biosynthetic Pathway Results in Significant Changes in the Cell Growth Phenotypes and Biosynthesis of Secondary Metabolites of Monascus purpureus
Source: J Fungi (Basel). 2022 Aug 27;8(9):910. doi: 10.3390/jof8090910 (PMC9503372; doi:10.3390/jof8090910)
Supplement: Supplementary file 1 [file jof-08-00910-s001.zip › Figure S2.pdf]

Figure S2. Comparison of the sensitivities of the wild type *M. purpureus* LQ-6 and the mutant strain *M. purpureus* Δ5162 cultured on PDA medium in respond to lactic acid (A), ethanol (B), H_2O_2 (C) and NaCl (D).

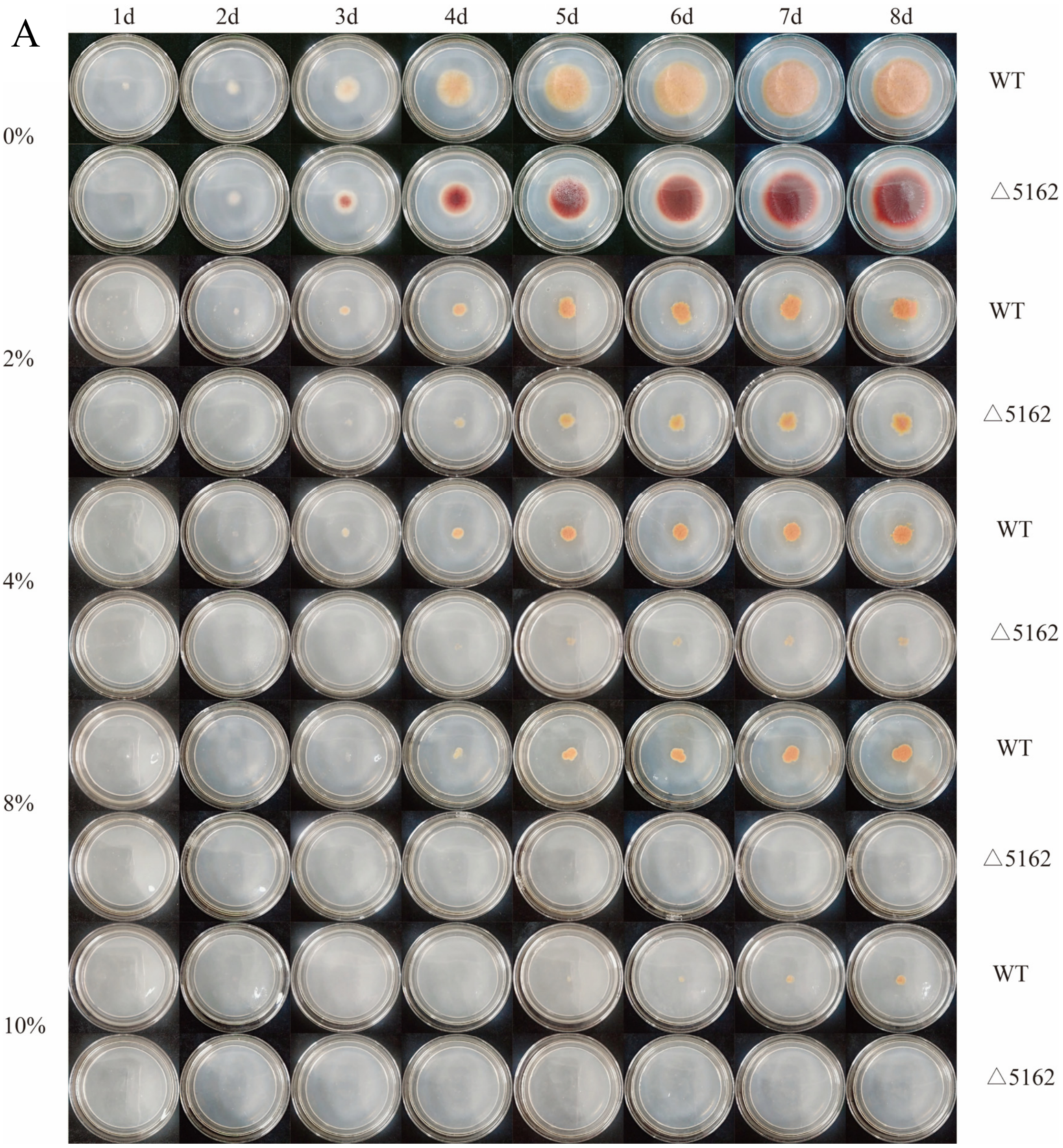

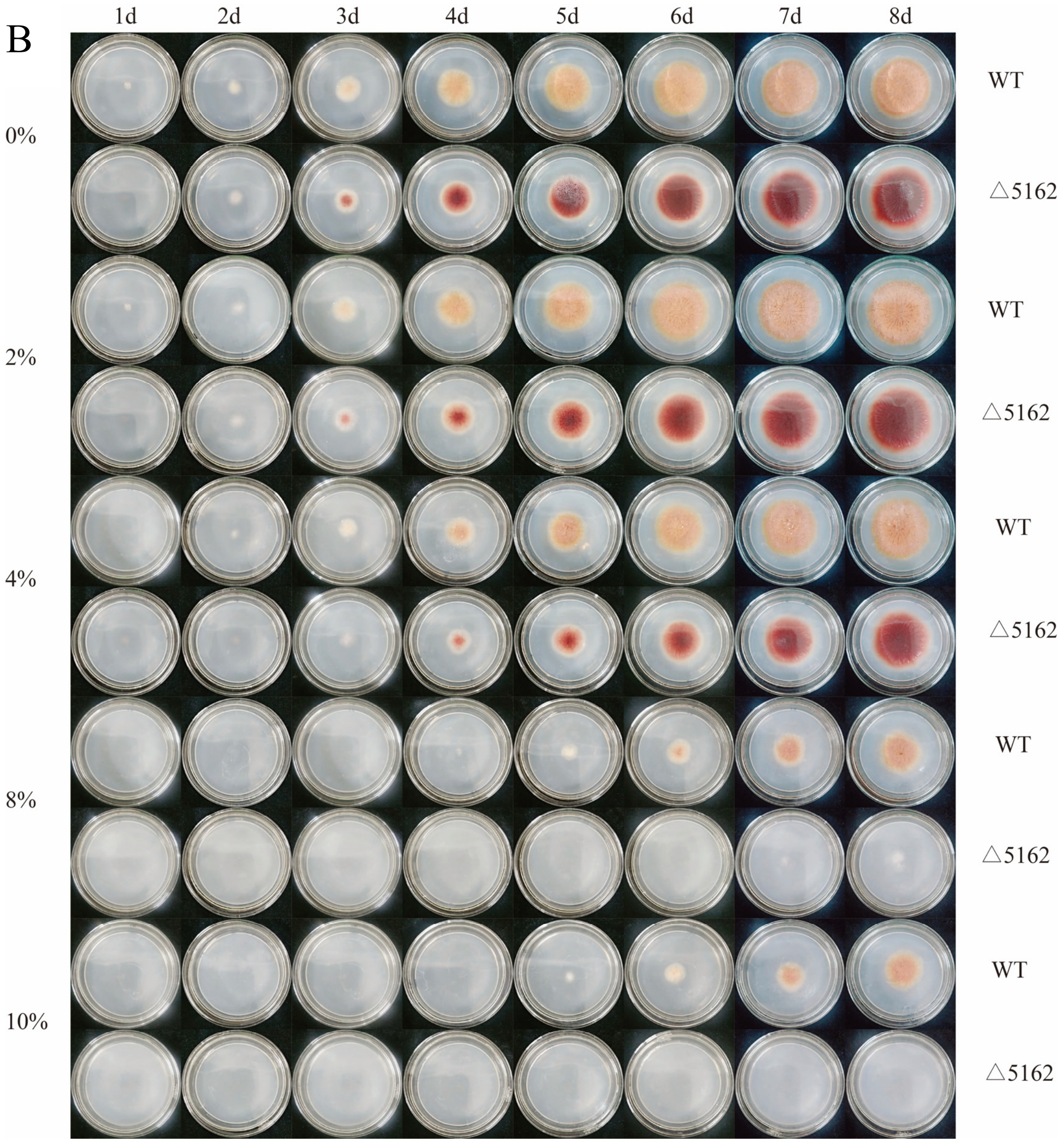
B
1d
2d
3d
4d
5d
6d
7d
8d
0%
2%
4%
8%
10%
WT
△5162
WT
△5162
WT
△5162
WT
△5162
WT
△5162

C

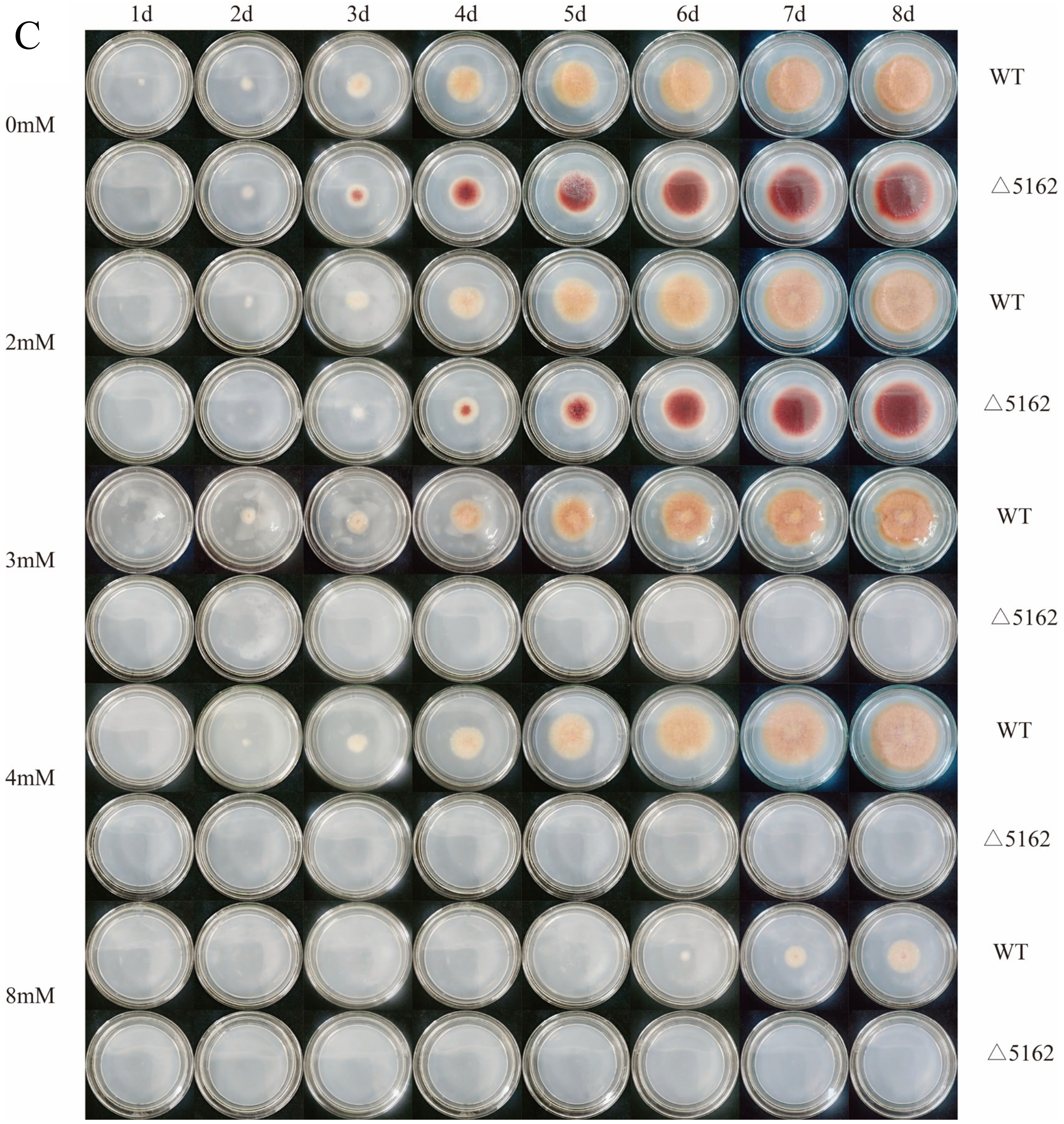
1d
2d
3d
4d
5d
6d
7d
8d
0mM
2mM
3mM
4mM
8mM
WT
△5162
WT
△5162
WT
△5162
WT
△5162
WT
△5162

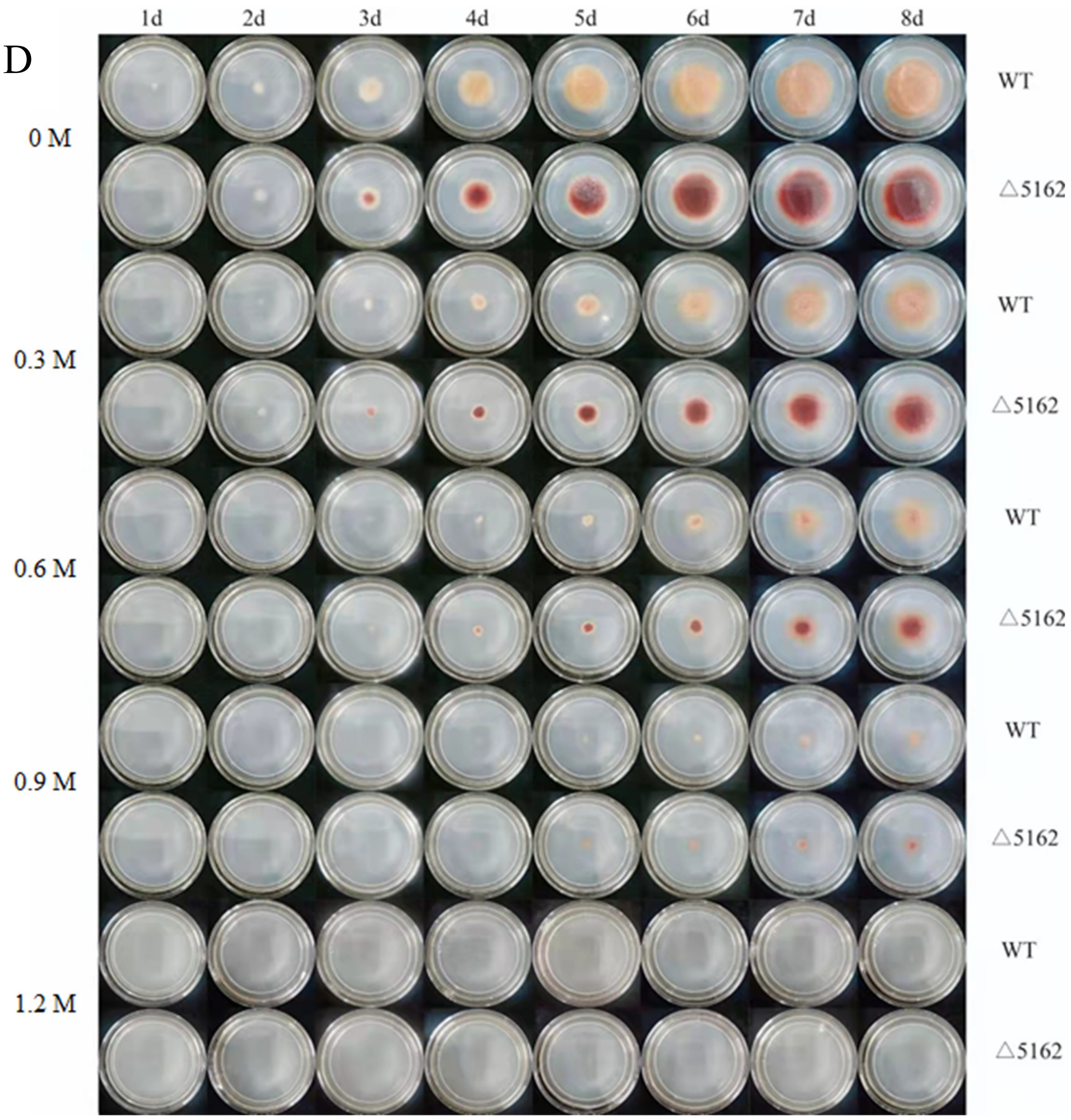
D
1d
2d
3d
4d
5d
6d
7d
8d
0 M
0.3 M
0.6 M
0.9 M
1.2 M
WT
△5162
WT
△5162
WT
△5162
WT
△5162
WT
△5162
