# Supplementary material for: Disruption of the Chitin Biosynthetic Pathway Results in Significant Changes in the Cell Growth Phenotypes and Biosynthesis of Secondary Metabolites of Monascus purpureus
Source: J Fungi (Basel). 2022 Aug 27;8(9):910. doi: 10.3390/jof8090910 (PMC9503372; doi:10.3390/jof8090910)
Supplement: Supplementary file 1 [file jof-08-00910-s001.zip › Table S1.pdf]

Table S1. The plasmids and strains used in the study.

Plasmid/ Strains	Relevant characteristics	Reference
**Plasmids**		
pXS-G418	Gene deletion vector	Presented
pBARGPE1-Hygro	Gene expression vector	Presented
pXS-5162	Derived from pXS-G418, with the 5162-deleted cassette added	This study
pBA-5162	Derived from pBARGPE1-Hygro, with the *chs 6* fragment added	This study
**Strains**		
E. coli DH5α	Cloning	Purchase
M. purpureus LQ-6	Wild-type strain	*Monascus. purpureus*
M. purpureus Δ5162	Knocking out gene *chs 6* in *M. purpureus* LQ-6	This study
M. purpureus cp-5162	Complementation expression of gene *chs 6* in *M. purpureus* Δ5162	This study
