# Supplementary material for: Disruption of the Chitin Biosynthetic Pathway Results in Significant Changes in the Cell Growth Phenotypes and Biosynthesis of Secondary Metabolites of Monascus purpureus
Source: J Fungi (Basel). 2022 Aug 27;8(9):910. doi: 10.3390/jof8090910 (PMC9503372; doi:10.3390/jof8090910)
Supplement: Supplementary file 1 [file jof-08-00910-s001.zip › Table S2.pdf]

Table S2. The primers used in the study.

Primer	Sequence (5'→3')	Reference
5162-up-F	ATAAGCTTGATATCGAATTCCTCCTCT CGGAAAAAGTCCC	This study
5162-up-R	ATACTAGTGGATCCCCCGGGATGACG GTAAGGCTTACAGT	This study
5162-dn-F	CCGCCACCGCGGTGGAGCTCTGATCTT TTGTTTGGGCCTTGA	This study
5162-dn-R	TTTGCTGGCCTTTTGCTCACATGTCACT TGTATCGGCATCCACC	This study
5162-T-F	ACTGTAAGCCTTACCGTCAT	This study
5162-T-R	TAGAGGCGACGGGATCCAGA	This study
pBA-5162-F	ACTCTAGAGGATCCCCCGGGATGGCG AATCGCTATTCTACC	This study
pBA-5162-R	GCTTGATATCGAATTCCTCACATGTTC GCTAGTTCA	This study
5162:cp-T-F	AGAAGTATGCAAAGCATGCG	This study
5162:cp-T-R	AACGTCATGCATTGCAGATG	This study
