# Supplementary material for: Disruption of the Chitin Biosynthetic Pathway Results in Significant Changes in the Cell Growth Phenotypes and Biosynthesis of Secondary Metabolites of Monascus purpureus
Source: J Fungi (Basel). 2022 Aug 27;8(9):910. doi: 10.3390/jof8090910 (PMC9503372; doi:10.3390/jof8090910)
Supplement: Supplementary file 1 [file jof-08-00910-s001.zip › Table S3.pdf]

Table S3. The germination rates (GR) of the conidia of the two strains *M. purpureus* LQ-6 and Δ5162 on the PDA medium at 30°C in the dark.

Strains	The theoretical number of colonies	The effective number of colonies-2 d	The effective number of colonies-5 d	GR-2 d	GR-5 d
M. purpureus LQ-6	120	106±3	110±3	89±2%	91±2%
M. purpureus Δ5162	80	18±3	18±4	22±3%	22±5%
