# Supplementary figures and images for: Disruption of the Chitin Biosynthetic Pathway Results in Significant Changes in the Cell Growth Phenotypes and Biosynthesis of Secondary Metabolites of Monascus purpureus
Source: J Fungi (Basel). 2022 Aug 27;8(9):910. doi: 10.3390/jof8090910 (PMC9503372; doi:10.3390/jof8090910)

**Figure S1.** Construction process of gene *chs6* deletion and complementation plasmids pXS-5162 and pBA-5162.

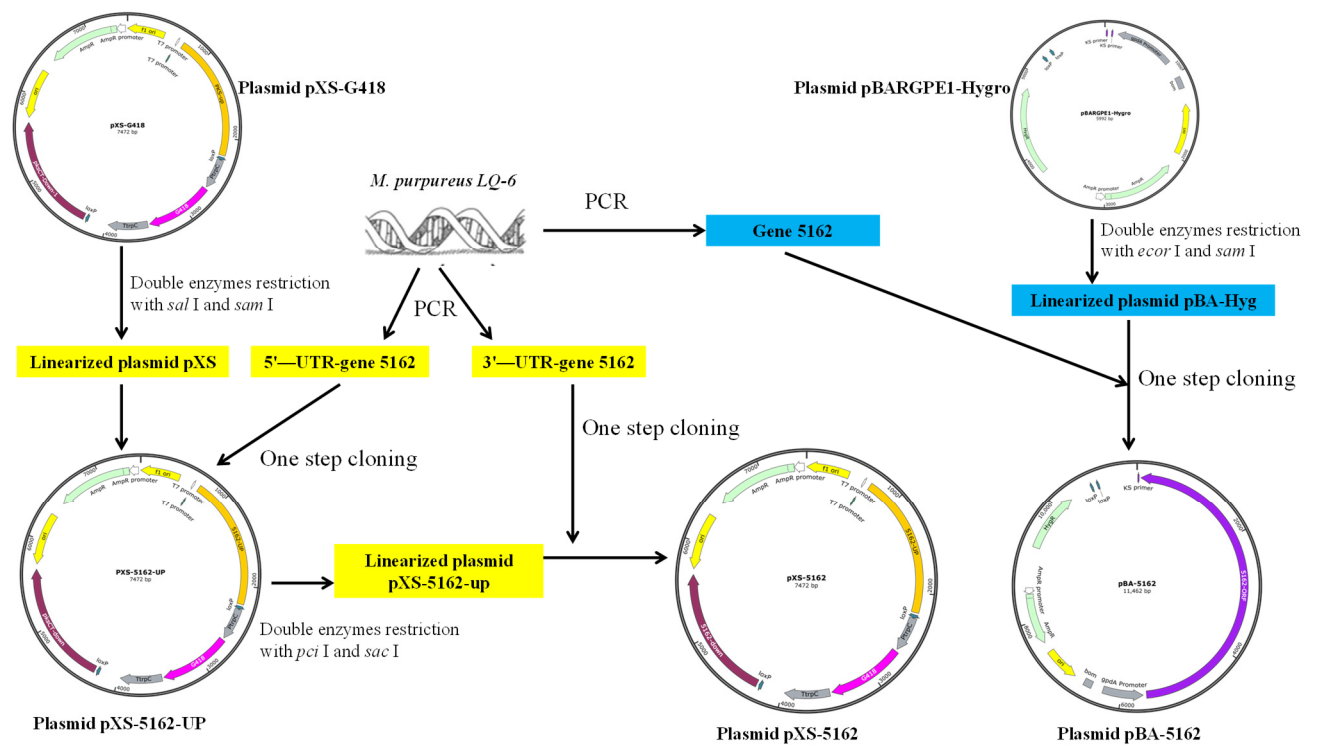

Supplement: Supplementary file 1 [file jof-08-00910-s001.zip › Figure S1.pdf]
